# Euthanasia in Dementia: A Narrative Review of Legislation and Practices in the Netherlands and Belgium

**DOI:** 10.3389/fpsyt.2022.857131

**Published:** 2022-06-02

**Authors:** Radboud M. Marijnissen, Kenneth Chambaere, Richard C. Oude Voshaar

**Affiliations:** ^1^Department of Psychiatry, University Medical Center Groningen UMCG, Groningen, Netherlands; ^2^The End-of-Life Care Research Group, Ghent University Hospital, Ghent, Belgium

**Keywords:** euthanasia, physician assisted dying, review, dementia, end-of-life, euthanasia law

## Abstract

Euthanasia was first legalized in the Netherlands and Belgium in 2001 and 2002, respectively. Currently they are among the few countries that also allow euthanasia on the basis of dementia, which is still considered controversial, both from a scientific and societal perspective. To date, euthanasia in dementia constitutes a small proportion of all Dutch and Belgian euthanasia cases. However, instances are rising due to a growing awareness among the general public about the possibilities of a self-chosen end-of-life and the willingness among medical professionals to perform euthanasia in individuals diagnosed with dementia. In both countries euthanasia is allowed under strict conditions in patients with dementia and decisional capacity regarding euthanasia, while in the Netherlands an advance euthanasia directive can also replace an oral request for euthanasia in those with late-stage dementia. Judging euthanasia requests from patients with dementia is complex and the assessment of the due care criteria (especially those related to decisional capacity and unbearable suffering) requires caution and great care. In this narrative review, we reflect on the legal regulation, clinical guidelines and societal debate regarding euthanasia in dementia in the Netherlands and Belgium. By discussing the 20 years of experience with the ethical dilemmas and controversial aspects surrounding this delicate topic, we hope to inform the preparation or implementation of new legislation on euthanasia in dementia in other countries.

## Introduction

At the start of the new millennium, the Netherlands and Belgium (also referred to as the Low Countries) were the first countries to legalize euthanasia and physician-assisted dying by adopting the Termination of Life on Request and Assisted Suicide Act and the Act on Euthanasia, respectively (1, 2). The difference between euthanasia and assisted dying depends on the person performing the final act that causes the death. Euthanasia is the active termination of life by another person at the request of the individual, while in assisted dying the patients themselves ingest a lethal substance supplied by another person unaided and with the explicit intention to end their life. For reasons of readability we will use the term euthanasia for both events (3). Euthanasia remains a punishable offense and can only be carried out by physicians under strict conditions and when all of the due care criteria and procedural requirements as laid down in the legislation are met (1, 2). It goes without saying that in the Low Countries many still have a negative view of people who choose to end their lives voluntarily and helping someone to die or to perform euthanasia is considered even more controversial (4).

Internationally, euthanasia legislation in the Netherlands and Belgium is still deemed progressive as the (chronic) disease does not necessarily result in death within the near future, including dementia and psychiatric disorders. Aside from these Low Countries, euthanasia for patients diagnosed with dementia is only permitted in Canada (5)^1^, Luxembourg, and Columbia (6)^2^ provided the individual has decisional-making capacity when lodging the request. In Switzerland, not euthanasia but only physician assisted suicide is permitted (6)^3^.

In the Low Countries euthanasia on the grounds of a dementia diagnosis clearly remains a controversial topic, both scientifically and societally (3). Nevertheless, the first Dutch case of euthanasia in a person with early-stage dementia in 2004 received hardly any public attention, while recently the so-called “coffee euthanasia”-case based on the advance euthanasia directive of a 74-year-old woman with late-stage dementia did fuel a heated public and legal debate that is still ongoing (for more details about the case, see **Box 5**). In Belgium the progression of events was similar; in 2004 the first euthanasia in early-stage dementia went virtually unnoticed, but the case of Hugo Claus, the widely acclaimed Belgian novelist, who was granted euthanasia in the early stages of his dementia in 2008 received considerably more attention in the Belgian media.

Persons living with dementia face the loss of their identity and social roles resulting from the gradual progression of deficiencies in their ability to think and function (7). They particularly fear being admitted to a nursing home, being unable to recognize loved ones and losing awareness of social norms (7). Many people dread the prospect of being bereaved of their defining characteristics and dignity through dementia and would prefer a timely death over having to live through the progressive stages of dementia (8). For those patients already living with dementia it is crucial to maintain control over their lives as long as possible, where discussing end-of-life decisions with a physician is considered highly meaningful (9). Being diagnosed with dementia is very difficult to face because disease-modifying therapies are still lacking and for most patients euthanasia is still one of the most sensitive subjects especially in this context, daunting many health professionals (3). The public debate in the Netherlands and Belgium focuses on the interpretation of the legal criteria for euthanasia, especially those that define unbearable suffering, decisional capacity, and the status of the advance euthanasia directive (AED) of patients with late-stage dementia (10, 11). AEDs are only covered in Dutch legislation and will be discussed in detail later in this review.

In this narrative review, we will describe the legal regulation of euthanasia with a special focus on euthanasia in dementia, its epidemiology and practices, current clinical guidelines and the societal debate in the Netherlands and Belgium, while critically reviewing the controversial and challenging aspects of its implementation.

## Euthanasia Legislation in the Netherlands and Belgium

In the Netherlands, the Termination of Life on Request and Assisted Suicide Act was adopted in 2001 and in 2002 the Belgian parliament approved the Act on Euthanasia, making them the first countries to formally legalize euthanasia (1, 2). Since both Acts place no restrictions on the nature (physical or psychological) of suffering or life expectancy, euthanasia in dementia is allowed under specified strict conditions. See Box 1 and Box 2 for the due care criteria stipulated in the Dutch and Belgian act, respectively.

Box 1 **Due care criteria termination of life on request and assisted suicide act—the Netherlands.**

Section Euthanasia Legislation in the Netherlands and Belgium of the Termination of Life on Request and Assisted Suicide Act provides medical due care criteria that must be met for euthanasia to be permitted and requires that the physician who performs the euthanasia:

- be convinced that the patient's request is voluntary and well-considered
- be convinced that the patient's suffering is unbearable with no prospect of improvement
- to have informed the patient about their situation and prognosis
- to have come to the conclusion, together with the patient, that there is no reasonable alternative in the patient's situation
- to have consulted at least one other independent physician, who must examine the patient and give a written opinion on whether the due care criteria have been met
- to have exercised due medical care and attention in terminating the patient's life or assisting in their suicide

Section Euthanasia Legislation in the Netherlands and Belgium (2) of the Act states “If a patient aged 16 or over who is no longer capable of expressing their will, but before reaching this state was deemed capable of making a reasonable appraisal of their own interests, has made a written declaration requesting that their life be terminated, the physician may comply with this request. The due care criteria in subsection 1 apply mutatis mutandis.”

________

Minister van Volksgezondheid, Welzijn en Sport (1).

Box 2 **Due care criteria Euthanasia act—Belgium.**

Physicians are allowed to perform euthanasia without committing a crime if they meet due care criteria and procedures (Art 3 No 1). They must ensure that:

- the patient has attained legal age or is an emancipated minor, is legally competent and conscious at the time the request is made;
- the request is voluntary, well-considered and repeated and not the result of any external pressure
- the patient must have a medical condition without prospect of improvement
- the patient must be experiencing constant and unbearable physical or psychological suffering that cannot be alleviated. This suffering should result from a serious and incurable disorder caused by illness or accident
- the patient is informed about the state of their health and life expectancy, the therapeutic measures that can still be considered, as well as the availability and consequences of palliative care
- assessment of the physician with the patient if all criteria are met
- the request was discussed with the treatment team
- consultation with an independent physician and, in case of a non-terminal phase, with a psychiatrist or specialist in the condition that prompts request for euthanasia and a waiting period between the written request and the execution of at least 1 month
- the request for euthanasia was written by the patient

________

Belgisch Staatsblad (2).

Although largely similar, the two Acts do differ with respect to several essential elements. Highlighting similarities and differences, Table 1 summarizes the essential components of the two Acts regarding a euthanasia request, advance directive, decision-making capacity, patient characteristics and clinical condition, and procedural aspects (1, 2).

**Table 1:** Differences and similarities in euthanasia in the Netherlands and Belgium.

	**The Netherlands**	**Belgium**
**Request**		
Voluntary, well-considered request	Voluntary and well-considered.	Voluntary, well-considered and not the result of any external pressure.
Repeated request	Not necessary.	Repeated request is necessary.
Written request	Not necessary.	Written request, dated and signed by the patient (valid for 5 years).
Informed consent	Informed about the state of their health and life expectancy, therapeutic measures that can still be considered, and the availability and consequences of palliative care.	Informed about the state of their health and life expectancy, therapeutic measures that can still be considered, and the availability and consequences of palliative care.
**Patient and clinical condition**		
Age	Of legal age (≥18 years), or in coordination with parents (16 or 17 years), or with permission of parents (age 12–15 years).	All ages, if decisionally competent when making the request.
Underlying condition	Medical condition with no possibility of improvement.	A serious and incurable disorder caused by illness or accident with no possibility of improvement.
Suffering	Unbearable.	Constant and unbearable physical or psychological suffering that cannot be alleviated.
Life expectancy	Not relevant	Not relevant
Irreversible coma / unconsciousness	No possibility because suffering has ended.	Possible based on written advance directive when decisionally competent.
Decisional capacity	Possible based with an advanced written directive (if still suffering unbearably).	No possibility because patients have to confirm their request before euthanasia (except coma).
**Procedural aspects**		
Professional who makes the decision	Responsible physician	Responsible physician
Examination by responsible physician	No requirements	Multiple appointments spread over a reasonable period
Conscience clause	Not included in the Act	Included in the Act; explanation in 7 days
Referral obligation	No requirements	Mandatory if the physician does not perform euthanasia
Family members	No requirements	Responsible physician is responsible for ensuring the patient has had the opportunity to discuss his request with all family members.
Independent consultation	An independent physician must evaluate the patient in person to confirm the following due care criteria: (1) well-considered and voluntary request, (2) unbearable suffering with no possibility of improvement, (3) well-informed about current situation and all treatment/palliative possibilities, and (4) absence of reasonable alternatives.	An independent physician must evaluate the medical records and the patient in person to evaluate the presence of unbearable physical and/or psychological suffering that cannot be alleviated.
Non-terminal phase/illness	No additional requirements	A second physician, i.e., an independent psychiatrist or specialist in the condition prompting the euthanasia, should be consulted, and a waiting period of 1 month is required between the written request and the euthanasia
Medical file	No requirements by euthanasia Act	By Act: keep medical file
Advance directive	Written when decisionally competent. Other due care criteria still apply, i.e., unbearable suffering with no possibility of improvement, well-informed about current situation at time of writing advanced directive, and absence of reasonable alternatives.	Only relevant for unconscious patients. Must be written before coma, in the presence of two witnesses, at least one of whom has no material profit
Performing medical euthanasia	Have exercised due medical care and attention in terminating the patient's life or assisting in his suicide.	Not passed into Act.
Declaration and review	Federale Controle- en Evaluatiecommissie Euthanasie (FCEE)	Regionale Toetsingscommissies Euthanasie (RTE)

*Minister van Volksgezondheid, Welzijn en Sport (1), Belgisch Staatsblad (2), and Handbook Psychiatry in end-of-life (3)*.

As can be deduced from Table 1, the Dutch Act is less explicit than its Belgian counterpart. While in Belgium a request for euthanasia must be repeated and accompanied by a written, dated and signed declaration, in the Netherlands a single oral request suffices. Nevertheless, in daily Dutch practice most, if not all, physicians consider the repetition of the request to be an important sign that the request is well-considered. Furthermore, in the Netherlands euthanasia is permitted when “unbearable suffering” is due to “a medical condition,” while in Belgium the “physical or psychological unbearable suffering” needs to be associated with “a serious and incurable disorder caused by illness or accident” (1, 2). The choice of general terms in the Dutch legislation was based on the idea that euthanasia and physician-assisted dying are always subject to current medical possibilities and social developments. In other words, the interpretation of the Act is contingent on the accumulation of jurisprudence, precluding the necessity to having to adapt the Act continuously (3). Despite the differences in wording, both Acts allow euthanasia on the basis of psychological suffering and in non-terminally ill individuals, which includes those with a psychiatric disorder, dementia, or accumulation of age-related diseases and conditions.

In both countries, the Act stipulates that attending physicians must request a second opinion from another physician, who then needs to see the patient independently and produce a written report stating whether the statutory due care criteria have been met (1, 2). From a legal perspective, this could be any physician provided that they don't know the person requesting euthanasia and is fully independent of the attending physician. In the Netherlands, the Royal Dutch Medical Association^4^ has established regional networks of independent, expert physicians known as SCEN physicians (SCEN: Support and Consultation on Euthanasia in the Netherlands) who have been specially trained and certified to perform these consultations. For continued SCEN certification, physicians need to perform at least 20 SCEN consultations every 5 years, to participate in intervision groups (at least 10 every 5 years), and earn pertinent continuing medical education (CME) credits (attending at least two relevant symposiums every 5 years). Also, any attending physician who receives a euthanasia request from a patient can ask a SCEN physician for advice and support. However, when the attending physician decides to proceed with the request, they need to contact another SCEN physician to conduct the actual SCEN consultation. In case a SCEN physician feels uncomfortable with the procedure given the underlying reason of the request, which is common when this concerns a psychiatric disorder or dementia, they can always ask a fellow SCEN physician to perform the consultation (12).

When the person seeking euthanasia suffers from a non-terminal illness, Belgian Act also requires two consultations. The successive physicians both verify the same statutory due care criteria independently, but the second physician must be an expert in the underlying condition that has led to the request. Furthermore, the Belgian Act stipulates a 1-month waiting period between the written euthanasia request and its execution. Neither of these two prerequisites apply in the Netherlands (1, 2).

One of the major differences between the Acts in the Low Countries concerns the role of advance euthanasia directives (AED) in case of late-stage dementia. In both countries, people can file a written advance directive with their general practitioner or consulting physician in which they state their wishes with regard to future treatments and end-of-life decisions. It is only in the Netherlands that an AED can replace an oral request when the decisional capacity of the patient becomes compromised (1). In such cases and provided that all other due care criteria are met at that moment, euthanasia is allowed in the absence of any current signs that contradict the wording or intention (see text Box 5) of the advance directive. Especially the “unbearable suffering” criterion is important here since euthanasia based on an AED can only be granted if both the attending and the SCEN physician are convinced that the patient's anguish is indeed unbearable (1, 12).

In Belgium, the person requesting euthanasia must have decisional capacity regarding euthanasia and repeat their request immediately before euthanasia is carried out as proof that the decision is voluntary and well-considered (13). With this prerequisite, the Belgian Act prohibits euthanasia in individuals with late-stage dementia and who have written AED when they lack decisional capacity at the time of the impending euthanasia (14). However, the Belgian Act does make an exception in case of an irreversible coma or vegetative state. The Dutch Act precludes this latter option because it cannot be determined whether an unconscious person is experiencing unbearable suffering; so not all statutory due care criteria are met (1, 2).

Even though euthanasia was legalized in the Lower Countries two decades ago, it is important to emphasize that no one has the legal right to demand euthanasia and physicians can never be compelled to perform it (1, 2). In general, most physicians are willing to perform euthanasia as such but not necessarily when their patient's request is associated with dementia or a psychiatric disorder. In these circumstances, it is good clinical practice in both countries for the attending physician to convey this directly to the patient and to then refer the patient to another physician. Furthermore, the Dutch and Belgian euthanasia Acts came about only after decades of heated public and political debate, which debate is still ongoing. Today, new discussions mostly focus on expanding the indications for euthanasia (15), but thus far proposals to change the Belgian Act to allow for euthanasia in advanced dementia have been unsuccessful.

## Applying Statutory Due Care Criteria in Dementia

In 2020, an American research group published a direct content analysis of 75 Dutch cases that had been published online by the Dutch Euthanasia Review Committees (in Dutch Regionale Toetsingscommissies Euthanasie RTE) between 2011 and 2018. They concluded that four out of 16 euthanasia cases based on an AED did not meet the statutory due care criteria, in particular the criterion of unbearable suffering (16). Kim et al. (17) concluded that individuals with early-stage dementia appear to be implicitly deemed decisionally competent, while in the Mangino study 15% of this group of people seeking euthanasia had been judged to be decisionally incompetent by at least one physician (16). These contradictory findings appear to show at least partial subjectivity in the interpretation of this due care criterion (see also Box 1). However, firm conclusions cannot be drawn due to a variety of methodological issues (18). Firstly, the published cases constitute a highly selective sample since they were the most controversial cases and were specifically published because of their educational value and the new insights that might be drawn from them. Secondly, all of the cases were summarized with an emphasis on the learning points. For example, when the criterion of unbearable suffering was undisputed, this topic may have been described too concisely for a proper content analysis. Thirdly, euthanasia approval processes can sometimes evolve over many years. The suffering caused by dementia can fluctuate over that time; in an early stage of dementia, a physician may conclude that the person seeking euthanasia is suffering unbearably, but this might not appear to be the case in a later stage, or vice versa. This does not mean that there are two different opinions about the suffering, but rather is a sign that the disease can evolve over time. By the same token, a person with early-stage dementia may lack decisional capacity regarding their euthanasia request due to a psychotic episode, for instance, but may fully regain this capacity after remission of this episode. The inconsistency in these cases is therefore related to a transient mental state and not to different judgments of the physicians involved (18). Still, as will be discussed below, inconsistencies in the evaluation of due care criteria can never be fully excluded because the criteria are dimensional in nature and inherently partially subjective.

We will focus on three due care criteria that will illustrate the challenges they pose, i.e., that the request must be voluntary and well-considered, that there is unbearable suffering with no prospect of improvement, and that there is no reasonable alternative in the patient's situation that will alleviate the suffering (joint conclusion).

### Voluntary and Well-Considered Request

To be able to conclude that the euthanasia request is made voluntarily and after careful consideration, it is important to establish that the person making the request has sound decision-making abilities, especially if they have dementia. Decision-making capacity is a key component of informed consent to medical treatment and since cognition is the main determinant in decision making, dementia could potentially impair this ability (19). A review of 32 studies on decision-making capacity in patients with Alzheimer's dementia showed that (1) the abilities to express a choice and to provide some reasoning for that choice are often preserved in this population, even in those with severe-stage Alzheimer's, and that (2) the Mini Mental State Examination [MMSE, (20)] can assist in stratifying the risk that an individual with Alzheimer's dementia lacks this capacity (19), where it is presumed that those with an MMSE score <16 tend to be incapable of understanding and appreciating alternative choices and those scoring >24 generally considered to retain their decision-making abilities. The predictive value varies for those with scores in between. A person requesting euthanasia in Belgium must be found competent to make a voluntary and well-considered decision (4). This means that in Belgium euthanasia can only be granted in the early stages of dementia for the reasons outlined above. At this stage, people generally have a good understanding of their disease and most will be cognitively able to substantiate their decision and request for euthanasia (4), while this becomes more difficult with certainty in more advanced stages of dementia (16).

Since any assessment of a person's decision-making capacity is subjective to some extent, professional guidelines recommend using the criteria formulated by Appelbaum and Grisso (21, 22). The authors propose to examine a patient in regard to their ability to communicate a choice, to understand the relevant information, to appreciate the (medical) consequences of the situation, and to reason in regard to (treatment) choices. Since decision-making capacity will decrease gradually linearly in cases of dementia, a functional approach recognizes that abilities may fluctuate and differ for types of decisions over time. Accordingly, it is important to emphasize that the timing of the assessment of decisional capacity is crucial and that it should not be compromised by the patient's state of mind due to their suffering. The “Euthanasia Code 2018” (Code of practice) as formulated by the RTE in the Netherlands recommends this functional approach in the assessment of decision-making capacity (23). Moreover, if there is any doubt about the decisional capacity in persons with dementia regarding their request for euthanasia, all Dutch clinical guidelines strongly advise that a dementia specialist with expertise in decisional functioning be consulted (12). Nevertheless, prompted by the cases published by the RTE, the Dutch evaluation practices for euthanasia requesters with dementia have been criticized for not conforming to the functional model of decision-making abilities and for applying low thresholds (16).

Limited verbal expression, the timing of the request, and verification that the desire to die is authentic and free from external pressure are three aspects that can play a critical role in the assessment of decisional capacity in dementia (3). In regard to the first question, it is important to note that when the person requesting euthanasia is no longer able to express themselves verbally, for example due to an expressive aphasia, they do not automatically lack decision-making capacity. In these cases, the patient's history and the course of the disease, (recent updates of) AEDs, and information from relatives must be considered in the assessment (12).

With regard to the timing of the request, many people recently diagnosed with dementia will consider euthanasia in the early stages of the illness to be premature. Their quality of life may remain relatively good for quite some time, with death still being far away and manifestations of the condition being manageable (14, 24). All parties involved are then confronted with a complex medical-ethical dilemma. From a medical viewpoint, euthanasia in the early stages of dementia implies that someone's life is terminated earlier than desirable, but delaying the decision for too long may lead to a situation where decisional incapacity precludes the termination of their life (4). People living with dementia do not want to die while they are still cognitively competent and enjoying life but they dread the possibility of waiting too long and losing the capacity to make a rational decision and confirm their earlier wishes (25).

Lastly, the request for euthanasia must be voluntary and constitute an authentic wish of the person making the request. Physicians must always explicitly rule out the possibility that the request was made under pressure. Partners, relatives and sometimes society may (unintentionally) induce feelings of guilt in the person with dementia, causing them to see themselves as a burden to their family, their healthcare providers, and even to society at large (4, 26). Especially in late-stage dementia, physicians should be aware that partners and relatives might (unwittingly) project their own worries and feelings onto their loved one, which can drive them to decide to request euthanasia (27). In cases of advanced dementia, when the request hinges on an AED, the physician must convince themselves that there are no indications against performing euthanasia, such as clear signs that the person involved no longer wishes to end their life. The Supreme Court in the Netherlands recently ruled that an AED should be interpreted according to the intention of the person requesting euthanasia and that physicians should consider all relevant circumstances rather than relying solely on the literal wording of the AED. In other words, an advance directive gives scope for interpretation [(28); also see Box 5].

### Unbearable Suffering

Suffering, and whether it is unbearable, is a strongly subjective experience, where the perspective of the person requesting euthanasia should take priority and be weighted most heavily (29). Suffering can be either psychological or physical and can be caused directly by the dementia, as well as by associated conditions. Dementia in and of itself does not necessarily lead to unbearable suffering, and the severity of suffering often fluctuates over time (29), and patients' thoughts about dementia and quality of life may also change during the course of the illness (30).

In the early stages of dementia there will be a gradual loss of functions, which is when people will realize that this process is progressive. In this phase, they may come to suffer from an overwhelming fear of the decline in their cognitive abilities, the negative impact on their autonomy and dignity, of becoming a burden to others and of having to be admitted to a nursing home (4, 31). To determine whether this suffering is genuine and unbearable, it is vital that the personality and life history of the requester be taken into account (3). For example, the fear of an undignified existence in the later stages of dementia may weigh more heavily when the attending or consulting physician comprehends that during the requester's life autonomy played a leading role or when they learn that the requester had cared for a family member who suffered unbearably from very severe behavioral and psychological symptoms of dementia.

Assessing the extent of suffering in late-stage dementia often is even more complex. According to the Dutch statutory due care criteria, the suffering must be present at that actual moment. To arrive at a decision, the physician(s) should talk extensively with the patient and observe them for a longer period of time at different times of the day, and obtain information from their partner and relatives and from medical records (12). Accordingly, in Dutch and Belgian clinical practice, the assessment is generally conducted by a multidisciplinary team.

### Reasonable Alternatives

Lastly, in late-stage dementia it can be similarly challenging to determine whether the “reasonable alternatives criterion” is satisfied because this has to be established *together with the patient* after they have been informed about the current situation, the diagnosis and prognosis (25).

## Late-Stage Dementia and the Written Advance Euthanasia Directive in the Netherlands

As mentioned above, in the Netherlands, an advance euthanasia directive can replace an oral request for euthanasia. When first diagnosed with dementia, people often formulate an AED to ensure that their wishes are respected, since the progression of dementia will inevitably affect their decision making-capacity regarding euthanasia (32). According to the Dutch Euthanasia Code of Practice (23), an AED must be drawn up when the writer is decisionally competent and must specify the future circumstances in which the writer would desire euthanasia also when they are no longer deemed to be decisionally competent. Although the Act allows the AED to be interpreted by the physician based on accumulated information (see also Box 5), it is desirable for the writer to indicate the specific circumstances in which the request should be acted upon as clearly as possible, most particularly, the circumstances or medical conditions that would be judged as unbearable and would justify euthanasia (23).

Both the person drafted the AED and their relatives may have high, often unrealistic, expectations. It is therefore important for treating physicians to explain the purpose of the directive to all parties concerned from the beginning (12, 33). They need to understand that a physician is not obliged to act on the AED, because having a directive does not automatically mean that all statutory due care criteria have been met. Many may find it difficult to imagine what they might experience once their cognitive abilities have declined and what their needs will be in the later stages of dementia, when end-of-life decisions can change as dementia progresses (34). Physicians will inevitably be faced with an ethical dilemma when the euthanasia request is founded on an AED. Should they grant the wishes of the “then self” (i.e., the self at the time of writing of the AED) or of the “now self” (i.e., the person's apparent interests, as confirmed by proxies acting on behalf of the person with late-stage dementia). For Dutch Act, the then self and the now self are the same person, rendering AEDs legally valid (35).

Since decision-making capacity will become compromised in all people living with dementia, Alzheimer's Nederland, the Dutch Alzheimer's Society, advocates discussing the AED in the context of advance care planning (ACP) as soon as possible after the diagnosis. Professional guidelines strongly recommend that AEDs be updated regularly (12, 16, 18). Although this is not mandatory, the older the AED is, the more doubt there may be as to whether the directive still reflects the current wishes of the requester. It is also advisable to involve loved ones and others at as early a stage as possible, since they may then be helpful in supporting the interpretation of the AED if necessary. Be that as it may, it must be made clear from the beginning that the decision of whether or not to perform euthanasia corresponds solely to the physician in interaction with the person requesting euthanasia; spouses, partners or relatives have no decision-making power from a legal standpoint (1).

In cases of late-stage dementia, when an AED is taken in lieu of an oral request, the due care criteria apply mutatis mutandis (Minister VWS, 2001), implying that they must be assessed while taking the present situation into account (e.g. limited communication with the requester) (23). In order to ascertain the current situation of the person requesting euthanasia, besides observing their behaviour closely, scrutiny of the available medical files and the advance directive itself, consultation with other (health) professionals involved in the person's care, and targeted dialogues with stakeholders (partner, family, caregivers, healthcare proxies) are most informative. All due care criteria must be met, which could mean that if suffering is not deemed to be apparent, the request for euthanasia cannot be granted even in the presence of a crystal-clear AED (35). In the literature, however, this stipulation is being debated, with some arguing that in the case of late-stage dementia, the AED should (morally) take precedence, even when the person involved is not suffering noticeably (35). Others contend that ‘the Dutch practice' of using AEDs as a decisional means to implement a patient's initial wish to die may hinder end-of-life care (25, 36).

## Euthanasia Review Committees

In the Netherlands and Belgium physicians are required by Act to report each euthanasia case for formal review (1, 2). The euthanasia review committees, i.e., the Federal Control and Evaluation Committee for Euthanasia (FCECE) in Belgium and the Regional Review Committees on Euthanasia (RTE) in the Netherlands, subsequently assess each case to determine whether the physician has acted in accordance with the statutory due care criteria and provide summary determinations to monitor the application of the Act. These review procedures ensures that cases are assessed consistently and transparently, which is important because physicians who fail to fulfill the due care criteria may be prosecuted (37).

Moreover, the Belgium FCECE publishes biannual reports (Federale overheidsdienst) and the Dutch RTE publishes its annual reports online (RTE, annual reports) when the cases will provide new insights or highlight new issues. These publications assist physicians and magistrates to uniformly interpret the Act.

As part of its educational mission, the review committee (RTE) has published a code of practice, updated in the Euthanasia code 2018, that explains how the review procedures work in practice and how the RTEs interpret the due care criteria (23). This code of practice aims to harmonize the judgments of the review committees in the Netherlands and serves as a guideline for physicians regarding the essential procedures and interpretation of the Act.

## Epidemiology of Current Practice

In the Netherlands and Belgium, a total of 290,000 and 193,000 people, respectively, suffer from dementia (38). While patients are increasingly seeking access to euthanasia (4, 24), thus far, euthanasia in patients with dementia has constituted only a small percentage of all cases in the Netherlands and Belgium (see Table 2). Most of the patients who receive euthanasia in the Netherlands (39, 40) and Belgium^5^ are suffering from the final stages of a malignant disease.

**Table 2:** Epidemiology of euthanasia based on dementia in the Netherlands and Belgium.

	**Overall mortality (all causes)**				**Dementia-related mortality**			
	**The Netherlands**		**Belgium**		**The Netherlands**		**Belgium**	
**Year**	Total number*	Euthanasia cases, *n* (%)**	Total number*	Euthanasia cases, *n* (%)**	Total number*	Euthanasia cases, *n* (%)**	Total number*	Euthanasia cases, *n* (%)**
2002	142,355	1,882 (1.3)	105,667	24 (0.0)	6,839	0 (n/a)	3,890	0 (0)
2003	141,936	1,815 (1.3)	107,068	235 (0.2)	7,046	0 (n/a)	4,242	0 (0)
2004	136,553	1,886 (1.4)	101,964	349 (0.3)	6,990	1 (<0.1)	3,936	2 (0.0)
2005	136,402	1,933 (1.4)	103,305	393 (0.4)	7,005	n/a	4,209	0 (0)
2006	135,372	1,923 (1.4)	101,614	429 (0.4)	7,688	6 (<0.1)	4,490	2 (0.0)
2007	133,022	2,120 (1.6)	102,060	495 (0.5)	7,267	n/a	4,692	1 (0.0)
2008	135,136	2,331 (1.7)	104,587	704 (0.6)	8,125	n/a	5,235	5 (0.1)
2009	134,235	2,636 (2.0)	104,509	822 (0.8)	8,204	12 (0.1)	5,370	7 (0,1)
2010	136,058	3,136 (2.3)	105,094	953 (0.9)	9,010	25 (0.3)	5,363	8 (0.1)
2011	135,741	3,695 (2.7)	104,247	1,133 (1.1)	9,150	49 (0.5)	5,437	10 (0.2)
2012	140,813	4,188 (3.0)	109,034	1,432 (1.3)	10,410	42 (0.4)	6,497	14 (0.2)
2013	141,245	4,829 (3.4)	109,295	1,807 (1.7)	12,617	97* (0.8)	6,672	13 (0.2)
2014	139,223	5,306 (3.8)	104,723	1,928 (1.8)	12,488	81 (0.6)	6,298	16 (0.3)
2015	147,134	5,516 (3.7)	110,508	2,022 (1.8)	13,861	109 (0.8)	7,162	20 (0.3)
2016	148,997	6,091 (4.1)	108,056	2,028 (1.9)	14,865	141 (0.9)	6,937	10 (0.1)
2017	150,214	6,585 (4.4)	109,629	2,309 (2.1)	15,460	169 (1.1)	7,441	14 (0.2)
2018	153,363	6,126 (4.0)	110,645	2,359 (2.1)	16,259	146 (0.9)	7,109	22 (0.3)
2019	151,885	6,361 (4.2)	108,745	2,656 (2.4)	15,750	162 (1.0)	n/a	26 (n/a)
2020	168,678	6,705 (4.0)	126,850	2,444 (1.9)	14,279	170 (1.2)	n/a	21 (n/a)

^*^The Netherlands: based on CBS Statline; Belgium: based on Statbel Belgium (all deaths) and Sciensano (dementia deaths); dementia related deaths coded as F00, F01, F02, F03, or G30.

^**^The Netherlands: based on annual reports of the Dutch RTEs; numbers of dementia-cases not explicitly reported in 2005, 2007, and 2008. Belgium: based on biannual reports of the Federal Control & Evaluation Committee Euthanasia (Dutch/French)-−2020 numbers based on press release dated 2 March 2021 (in Dutch). Euthanasia cases based on dementia in Belgium in the period 2002–2013 reported in: Dierickx et al. (41).

*n/a, not available*.

A nationwide survey (*n* = 5,361) in the Netherlands found that 11.2% of people who had died non-suddenly had requested euthanasia, and of these, 56% actually died by euthanasia or physician-assisted dying. These figures contrast with those of persons who died from dementia; only 2.1% of this subgroup had requested euthanasia, and of these “only” 43% died by euthanasia (42).

In the Netherlands and Belgium, the first patients received euthanasia based on a diagnosis of dementia in 2004 (see case 1; Box 3) and 2008, respectively. Since then, the absolute numbers have gradually increased. In 2020, in Belgium, a total of 22 persons received euthanasia based on a diagnosis of dementia^6^; see case 2 in Box 4. In the Netherlands, the absolute number of cases of euthanasia based on dementia more than tripled over the past decade, from 49 in 2011 to 170 in 2020. First, this corresponds with an increase from 1.3 to 2.5% of all euthanasia deaths (see Table 2). Secondly, the 170 persons with dementia who received euthanasia constituted 1.2% of the 14,279 persons who died from dementia in the Netherlands in 2020. Since there were no changes to the Acts and regulations, these increases should be interpreted as a growing social awareness of the possibilities for a self-chosen end-of-life in dementia and a growing willingness of medical professionals to perform euthanasia in patients with dementia (41). Nevertheless, it is four times less prevalent in comparison with the prevalence among all-cause mortality cases (see Table 2).

Box 3 **First case of euthanasia in (early-stage) dementia in the Netherlands (2004).**

X was a 65-year-old man who had been diagnosed with Alzheimer's disease 3 years earlier. The depressive symptoms that developed as a result of the diagnosis were successfully treated with antidepressants, and X also received day treatment in a local nursing home. X claimed to suffer unbearably because he could no longer function independently and as a result of “his future as a dementia patient.” Directly after the diagnosis, X reported to his physician that he did not want to go through the disease process and during the year prior to the euthanasia he had repeatedly asked the physician for assisted suicide.

His physician consulted an independent SCEN physician, who confirmed that the patient suffered greatly from his dependence on others, the awareness of his continued decline and loss of dignity, the loss of autonomy and self-esteem, and the knowledge that his situation would only get worse. Nevertheless, the SCEN consultant could not acknowledge that X's suffering was unbearable. Moreover, X's awareness of his deficits would diminish as his illness progressed, along with his suffering. Although the request for euthanasia had been consistently expressed over a long period of time, the SCEN consultant judged X's legal decisional capacity to be questionable because X was unable to demonstrate or comprehend consistent, coherent reasoning during the consultation. The consultant concluded that the due care criteria had not been met.

After the SCEN consultant's assessment, X's physician consulted three more experts: a psychologist, a geriatric physician and an geriatric psychiatrist. From the examinations that each of these experts conducted individually, it became clear that X was not depressed but that he wanted to maintain control over his life and that he was aware that with the progression of his Alzheimer's he would lose control. The three experts independently concluded that X was able to make a voluntary and well-considered request for termination of life and that he was aware of the consequences of his choice. Based on these evaluations, X's physician decided to grant the patient's request.

The Euthanasia Review Committee (RTE) concluded that although the SCEN consultant questioned the patient's mental capacity, the three experts consulted had individually concluded that X was indeed mentally competent and was well able to determine and substantiate his request. According to the committee, the physician had complied with the consultation requirement and, following the conflicting opinions of the SCEN consultant and the three experts, he had rightly assigned more weight to the opinions of the latter. The RTE concluded that on this basis the physician had been correct in deciding to agree to assisted suicide and that he had acted in accordance with the pertinent due care criteria.

Box 4 **A case of euthanasia in early-stage dementia in Belgium (2018/2019).**

Y was an 84-year-old man with early-stage Alzheimer's disease. In his active life, he had been the CEO of a multinational company. The diagnosis of Alzheimer's dementia was supported by a neuropsychological work-up, a brain MRI and a decreased concentration of amyloid beta-42 in cerebrospinal fluid. The initial treatment with donepezil had to be discontinued because of adverse events. The rivastigmine transdermal patch caused skin rashes at the application sites, but Y continued using the medication. He complained of memory disturbances and episodes of confabulations and observed that he was “mentally destroyed,” and hated the fact this had affected his quality of life so massively. The cognitive decline made him realize that his life had become meaningless. Y had witnessed this decline in his father and did not wish to go through the same process himself. Y no longer engaged in any meaningful daily activities, had largely lost his autonomy and had become dependent on his wife, who took excellent care of him. He had come to realize that his situation was hopeless and that there was no prospect of a cure or improvement. He wished this situation to end. For him, euthanasia was the only way to say goodbye to life in a dignified manner. His greatest fear was the possibility that his request for euthanasia would no longer be granted in light of the current legal provisions in Belgium and the impending progression of his cognitive dysfunction. His request for euthanasia was well-considered and had frequently and intensively been discussed with his wife and children. Both the general practitioner End-of-Life Information Forum (LEIF) physician and treating neurologist declared the request admissible and judged the patient to be decisionally competent.

In contrast to Belgium, Dutch Act allows for euthanasia in patients with late-stage dementia and decisional incapacity, in the presence of an advance directive in writing. However, this possibility is quite controversial among physicians and scientists (16), and is rarely used [Annual report, (37)]. In 2011, the Dutch RTE for the first time reported explicitly that a decisionally incompetent patient with late-stage dementia had received euthanasia. Since then the actual number of these patients has varied between 2 and 3 per year. These “low” figures of euthanasia in late-stage dementia can be explained by the ethical dilemmas physicians face, the public debate (25), as well as an ongoing debate on the interpretation of the Dutch Act.

## Medical Professional Guidelines on the Assessment of Euthanasia Requests

In both low countries, medical professional associations advise attending physicians to exercise caution and care in the case of a request for euthanasia in dementia, because it concerns a vulnerable population in which it is difficult to judge euthanasia (see below) (12). Although a second independent physician (beside the independent SCEN physician) is not required by Act in the Netherlands, the clinical guideline of the Royal Dutch Medical Society (KNMG) strongly recommends that a second independent physician should be consulted. This second independent physician must be an expert in the underlying condition and will have to determine whether the patient is mentally capable regarding the euthanasia request, whether the medical diagnosis is correct, and lastly, whether alternative treatment strategies to alleviate suffering have been overlooked. In the case of dementia, this should be a geriatric psychiatrist, neurologist, geriatrician, or elderly-care physician (12).

As discussed above, requests for euthanasia based on dementia are complex and pose several medical-ethical dilemmas. The practical professional guidelines therefore need to be developed further so that physicians respond properly, especially as the number of requests increases in Belgium and the Netherlands (41).

In the Netherlands, a clinical guideline and associated prerequisites for performing euthanasia have been developed under the auspices of the Royal Dutch Medical Association (KNMG). Prior to 2015, the guidelines were more conservative than the euthanasia Act itself (43). For example, the guideline indicated the prerequisite that patients must verbally confirm the request for euthanasia, even when there was an advance euthanasia directive. In 2020, the Supreme Court's ruling in the controversial “Coffee euthanasia” case (see case 3 Box 5) shed new light on the interpretation of the Dutch euthanasia Act.

Box 5 **A case of euthanasia in late-stage dementia in the Netherlands.**

In the Netherlands, the public debate on euthanasia intensified in 2017 after two Regional Review Committees concluded that a physician had not complied with the due care criteria in performing euthanasia on the basis of an advance euthanasia directive (AED) of a 74-year old woman with late-stage dementia. Both committees raised several concerns with the case, which included the limited wording of the woman's AED and the inappropriate use of premedication. The medical disciplinary court likewise determined that the physician had not complied with the statutory due care criteria and issued a written warning. The case became known as the “Coffee euthanasia case” because the physician had put midazolam in the patient's coffee without telling her.

In 2018, and for the first time since the introduction of the Termination of Life on Request and Assisted Suicide Act in 2001, the physician was prosecuted by the public prosecutor. The Criminal Court acquitted the physician in 2019, ruling she had in fact met all due care criteria, with this acquittal upheld in 2020 by the Dutch Supreme Court, adding that the wording of the AED should not merely have been taken literally since it left some room for interpretation. Also according to the Supreme Court, it is not mandatory to talk with and inform the patient about the procedure immediately prior to performing the euthanasia when the patient may not be able to understand what is being discussed and what is about to happen, and may even decompensate. Therefore, the Supreme Court did not endorse the Review Committees' concerns regarding the lack of communication and the absence of oral confirmation of the patient's wish to die; in this specific case pre-sedation was allowed since restlessness and agitation were to be expected (www.uitspraken.rechtspraak.nl). Although this ruling implies that in the Netherlands, an AED can replace an oral request when the requester has late-stage dementia, the prosecution of the physician did prompt concerns and anxiety among Dutch physicians regarding the possibility of prosecution after euthanasia (35), especially in individuals with late-stage dementia.

Following the Supreme Court's decision, the review committee adjusted its code of practice (23), updated version) and the Royal Dutch Medical Association (KNMG) published a new guideline about end-of-life decisions, with a special focus on euthanasia in dementia (12).

Four aspects of the code of practice [(23), updated version] have been adjusted: (1) there is room for interpretation of the advance euthanasia directive (AED; see below for further explanation of the advance directive); (2) the assessment of unbearable suffering with no prospect of improvement is reserved for physicians, so the RTE will judge this assessment with restraint; (3) in a patient with an AED in late-stage dementia who is decisionally incompetent, it is not necessary to discuss the euthanasia just before performing the euthanasia because this patient will not understand it; and lastly, (4) if restlessness, agitation or aggression are expected before performing euthanasia in a decisionally incompetent patient with late-stage dementia, pre-sedation without notification and consent of the patient may be indicated.

A comparable interpretation of the Act and a practical translation of the Supreme Court's decision can be found in the professional standards of the new guidelines of the Royal Dutch Medical Association (12):

- An advanced euthanasia directive (AED) must be discussed by the patient with the physician when the patient is decisionally competent.
- A physician must attempt to discuss the request for and performance of euthanasia, even if the patient is decisionally incompetent. If the patient expresses opinions that are contrary to the AED, then the due-care criteria are not met and euthanasia is not allowed.
- In judging the unbearable suffering, the physician must proceed cautiously and carefully. The suffering of the patient must be current, consistent, and observable.
- The reasonable alternatives to reduce suffering, like palliative care, changes in care, and medication, must be carefully considered. Preferably an expert should be consulted about this.
- There is room for some interpretation of the AED. The physician must consider all circumstances, and not only the literal terms of the AED. It is advisable to discuss the AED with relatives and caregivers to reach the best possible interpretation of the AED.
- According to the professional requirements, in addition to the independent physician that is required by Act (usually a SCEN-physician), at least one expert physician, such as a geriatric psychiatrist, neurologist or geriatrician, must also be consulted.
- If there are any indications that the patient will be restless, agitated, or aggressive when the euthanasia is performed, premedication is allowed.

Precise documentation is mandatory, with a description of all of the stages in the process (1). In addition to the clinical guidelines on the procedural aspects and interpretation of the Act, the KNMG has also developed Guidelines on the execution of euthanasia and physician-assisted suicide in collaboration with the Royal Dutch Society for Pharmacy (KNMP)^7^.

## Opinions About Euthanasia in Dementia in the Low Countries

In general, Euthanasia and Physician-Assisted Dying are increasingly regarded as an acceptable final option for patients suffering from chronic medical conditions with no prospect of improvement (44). Nevertheless, some researchers warn of a “slippery slope” concerning euthanasia and argue that the legal criteria can be and are overstretched, especially the criterion of incurability for patients with psychiatric disorders and in cases of so-called accumulation of age-related diseases and conditions (15).

Worldwide, healthcare professionals have more restrictive views toward assisted dying in dementia than the general population, patients, and caregivers (27). In the Netherlands, euthanasia in dementia is still controversial, with only 40% of physicians considering it acceptable to perform euthanasia in patients with early-stage dementia (45). Nevertheless, the debate in society focuses mainly on euthanasia in patients with late-stage dementia, especially on the determination of the due care criteria (11) and inconsistent or opposing wishes in an advance directive (46). In the Netherlands, 60% of the general population considers people with late-stage dementia eligible for access to euthanasia. This contrasts with only 24% of the general practitioners, 23% of clinical specialists and 8% of the elderly-care physicians working in nursing homes who consider it acceptable to perform euthanasia in people with advanced dementia (11). Dutch physicians consider direct communication with the patient about decisions on euthanasia to be very important. Performing euthanasia when the patient cannot confirm that the request is voluntary is a line many physicians refuse to cross (4, 47). The discrepancy between the general population and physicians can cause tension when physicians feel forced to perform euthanasia, and relatives are disappointed because “the final wish of their loved one” is not being respected. A qualitative study in the Netherlands showed that euthanasia requests in cases of dementia place a burden on general practitioners and elderly-care physicians. They feel pressured by relatives, patients and society and experience difficulties with interpretation of the Act, in addition to the ethical considerations (43, 48).

In Belgium, a small study of 17 physicians specialized in dementia showed that most of them (13/17) approved of the Act, but a majority (11/17) also did not agree to extend the Act to allow euthanasia based on an advance directive for patients with late-stage dementia (49). Fifty-six percent of Flemish general practitioners, especially the younger and non-religious physicians, agreed that the Act needed to be adjusted for patients with dementia. The general practitioners who disagreed with legalization of euthanasia in advanced dementia argued that quality of life cannot be judged reliably in this stage and that patients cannot repeat their will just before euthanasia is carried out (50).

## Expertise Centers in the Netherlands and Belgium

### Euthanasia Expertise Center (EE) in the Netherlands

The End-of-Life-Clinic (“Levenseindekliniek”) was founded in 2012 by the Dutch Right to Die Society (NVVE). In 2019 the name was changed into Euthanasia Expertise Center (“Expertisecentrum Euthanasie” of EE). According to EE's vision, every person with a request for euthanasia has the right, and should have the opportunity, to be examined by a physician to determine whether their request complies with the statutory due care criteria^8^. The EE gives information, ongoing training and education, advice, and concrete support, such as supervision of physicians during the euthanasia trajectories of their patients. Since a medical physician can never be forced to perform euthanasia in the Netherlands, the EE will provide euthanasia for all patients who request it and who meet the statutory due care criteria. The EE offers a safety net, especially for patients with complex requests for euthanasia, such as those based on a dementia.

The EE is not an actual physical clinic but rather is a network of 140 physicians and nurses throughout the Netherlands. Compared to national figures for the Netherlands, a relatively large number of patients with dementia, mental disorders, and an accumulation of age-related health deficits register requests for euthanasia with the Euthanasia Expertise Center, because general practitioners generally consider these cases to be complex or do not perform euthanasia in non-terminally ill patients as a matter of principle. As a result, the waiting-lists for these patient groups are increasing enormously (currently up to 2 years for patients with a mental disorders)^6^.

### The End of Life Information Forum and End of Life Forum in Belgium

An End-of-Life-Clinic like the Euthanasia Center of Expertise does not exist in Belgium. In 2003, the End of Life Information Forum (Levens Einde Informatie Forum, LEIF) and End of Life (EOL) Forum were founded in Belgium. Both consortiums focus on informing and educating patients, relatives and professionals. LEIF is an open initiative of people and associations that aims to achieve a worthy end of life for everybody; respect for the patient's desires is paramount.

Physicians can register themselves as LEIF-physicians, with the prerequisite they will comply with ongoing education and supervision requirements. Similar to the SCEN-physicians in the Netherlands, LEIF-physicians can be consulted as independent physicians, when a physician intends to perform euthanasia in one of their patients.

In addition, several centers of expertise have been founded in Belgium, including “Dignified End-of-Life Center of Expertise” (W.E.M.M.E.L.) in 2011, the End-of-Life Request Assessment Team (“Uitklaring levenseindevragen Team,” Ulteam) and Vonkel, Center for End-of-Life Questions in Ghent in 2015. These centers offer advice and support for patients, relatives and physicians with complex end-of-life questions, including euthanasia. Patients can contact the center in Vonkel and Ulteam for the assessment of their euthanasia requests. These centers receive a disproportionately high number of applications from patients with psychiatric profiles (including dementia) (51).

## Contextual Aspects of Euthanasia in Dementia

The gradual deterioration of cognitive and physical functioning in dementia confronts patients with many social and medical issues for which they must make very personal choices. In our opinion the (im)possibility of euthanasia in dementia should be discussed with the patient and relatives in the broader context of advance care planning and palliative and end-of-life care. Well-developed end-of-life care for patients with dementia should be attainable for all of them and is never incompatible with euthanasia.

### Advance Care Planning

Advance care planning (ACP) is a dialogue process that enables people to formulate important personal values and life goals, but also to think about the meaning and effect of a severe disease and to formulate preferences regarding future medical decisions (52). ACP encourages people to appoint a personal representative, establish personal preferences and discuss those preferences regularly with healthcare professionals and family members (52). Since cognitive decline continues during the process in people with dementia, individual preferences for health and end-of life care should be discussed before there is obvious deterioration. Regular establishment is important because the situation and decision-making capacity of patients with dementia change during the process. ACP has been proven to prevent futile interventions and provide comfort to patient with dementia, allow better access to palliative care and also relieve the burden on caregivers and reduce healthcare costs (53). The fear that the final stages of living with dementia will not expire with dignity (7) makes ACP especially important for patients with dementia. However, ACP is poorly implemented in persons with dementia worldwide (54, 55). This is a missed opportunity, because ACP results in care that is more closely aligned with the patient's personal preferences, greater satisfaction with the care, and also a postponement of hospitalization (56). The importance of ACP is endorsed in both the Netherlands and Belgium. The Dutch Alzheimer's Society^9^ and the Flemish Federation of Palliative Care (www.palliatief.be) recommend that the (im)possibility of euthanasia be discussed as soon as possible after dementia is diagnosed, in the broader context of ACP, meaning of life, palliative care and end-of-life care, to create the possibility for the patient and physician to share the process. A geriatric assessment can provide a good basis for a shared decision process (57), considering the preferences of the patient with cognitive impairment in decision making (58). In patients with advanced dementia, ACP should consider the wishes of the “then self” written in the ACP at the time the person had actually decisional capacity and the actual wishes of the “now self,” expressed non-verbally by the patient and interpreted by relatives and professionals (36).

### Palliative and End-of Life Care

It is important to state here that, first of all, professionals who care for patients with dementia must have skills for providing good end-of-life care. In addition to adequate technical-medical care, professionals should be able to meaningfully connect with patients and be able to explore their suffering and understand the narratives of the patient (59–62). A care approach that enhances dignity is proposed to reflect on the end-of-life of persons with late-stage dementia and the role of AEDs. The model begins with the experiences of vulnerability, followed by responding with a shared understanding of adequate care to ascertain dignity. In the dignity-enhancing care model, it is important to take a relational view of autonomy, to engage an understanding of the practice of Advance Care Planning (ACP) and to apply palliative care to persons with dementia.

## Conclusion and Future Directions

As the first countries to implement and track euthanasia and physician-assisted dying in patients with dementia, Belgium and the Netherlands provide 20 years of development and experience that could benefit other countries with new euthanasia legislation. Dealing with euthanasia requests in patients with dementia is highly complex because of the conflicting medical and ethical questions that they raise (32). Cases of euthanasia in dementia have increased significantly, but they still account for a small proportion of all dementia-related deaths and are also proportionally lower in comparison with the proportion of euthanasia among all-cause deaths.

Despite the long tradition, euthanasia in patients with dementia is still controversial in the Low Countries. As described in this paper, interpretation of the Act (the Netherlands) as well as efforts to expand the Act (Belgium regarding euthanasia in late-stage dementia) are still ongoing. For example, more research is warranted regarding uniform assessment of decisional capacity and unbearable suffering in the context of a request for euthanasia in dementia (16).

The discrepancy in the attitudes toward euthanasia in dementia of physicians and the general population sometimes causes tension in daily practice when the expectations of patients and their families may not be met (43, 48). Since the assessment of the request for euthanasia in persons with dementia and the performing of euthanasia is difficult for patients, their relatives, and the professionals, more research is needed into how to best support patients, relatives and also physicians during and after these processes. Also, nationwide education programmes about the (im)possibility of euthanasia in dementia may be needed. Both the general population and physicians probably need time to adapt to new ideas on euthanasia in vulnerable patients and hopefully their opinions will converge.

Discussing and informing patients about the (im)possibility of euthanasia in dementia should be part of advance care planning. From the patient perspective, the Dutch Alzheimer's Society advocates starting this discussion as soon as possible after dementia is diagnosed, as a beginning of a shared process. In the ACP process, repeated conversations between the (still competent) patient, the loved ones and the physician about all kinds of end-of-life decisions, including the (im)possibility of a self-chosen end-of-life, will help to improve end-of-life care for patients with dementia. To this end, it is important to recognize that well-developed dementia and palliative care should be attainable for all patients and is never incompatible with euthanasia. Nevertheless, there is no empirical data available as to how to properly implement these discussions as a part of ACP.

## Author Contributions

RM performed the literature search and wrote the first draft. RM, KC, and RO reviewed the literature, made a significant contribution to the final draft, and are the editors of the handbook, and also the authors of chapter 4.5, Euthanasia in dementia. KC added further information about euthanasia based on dementia in Belgium. KC and RO commented on the first draft. This paper is partly based on the Dutch Handbook Psychiatry in End of Life. All authors contributed to the article and approved the submitted version.

## Conflict of Interest

The authors declare that the research was conducted in the absence of any commercial or financial relationships that could be construed as a potential conflict of interest.

## Publisher's Note

All claims expressed in this article are solely those of the authors and do not necessarily represent those of their affiliated organizations, or those of the publisher, the editors and the reviewers. Any product that may be evaluated in this article, or claim that may be made by its manufacturer, is not guaranteed or endorsed by the publisher.

## Footnotes

^1^Canada: www.canada.ca/en/health-canada/services/medical-assistance-dying.html.

^2^Colombia: Colombia: www.minsalud.gov.Co/NormatividadNuevo/Resolución %201216%20de%202015.pdf (Spanish language).

^3^Switzerland: https://www.bj.admin.ch/bj/en/home/gesellschaft/gesetzgebung/archiv/sterbehilfe/formen.html.

^4^KNMG: https://Standpunt levenseinde - KNMG Standpunt Levenseinde (maglr.com).

^5^Federale overheidsdienst: https://fcee-cijfers-2020_persbericht.pdf (belgie.be).

^6^ https://www.euthanasiecommissie.nl/de-toetsingscommissies/uitspraken/~brochures/

^7^KNMP: https://www.knmp.nl/praktijkvoering/richtlijnen.

^8^Expertisecentrum Euthanasie: https://expertisecentrumeuthanasie.nl/wachttijd-door-grote-hoeveelheid-aanmeldingen/.

^9^Alzheimer's Nederland. www.alzheimer-nederland.nl.
